# A randomized, controlled, two-month pilot trial of stannous fluoride dentifrice versus sodium fluoride dentifrice after oxalate treatment for dentinal hypersensitivity

**DOI:** 10.1007/s00784-020-03275-8

**Published:** 2020-05-10

**Authors:** Chad J. Anderson, Gerard Kugel, Yuanshu Zou, Marco Ferrari, Robert Gerlach

**Affiliations:** 1 Department of Prosthodontics and Dental Materials, School of Dental Medicine, University of Siena, Siena, Italy; 2 DMD Inc., Fresno, CA USA; 3 Department of Comprehensive Care, School of Dental Medicine, Tufts University, Boston, MA USA; 4 The Procter & Gamble Company, Mason, OH USA

**Keywords:** Dentin desensitizing agents, Dentin sensitivity, Oxalates, Sodium fluoride, Stannous fluoride, Topical preparation

## Abstract

**Objectives:**

To compare the effects of a stannous fluoride dentifrice and a sodium fluoride dentifrice on dentinal hypersensitivity when used with an oxalate-based regimen combining in-office and at-home treatment.

**Materials and methods:**

In this single-center, randomized, controlled, double-blind, pilot clinical trial, 30 subjects were professionally treated at baseline with a 3% oxalate/potassium salt solution on up to two target teeth, then randomized 1:1 to either 0.454% stannous fluoride or 0.243% sodium fluoride overlabeled dentifrice. Both groups were given 6 sensitivity strips (3.14% potassium oxalate gel) and a soft, manual toothbrush. Subjects were permitted to apply strips on up to two teeth, up to three times per tooth, at home as desired throughout the study. Dentinal sensitivity (cold air blast challenge) was assessed at baseline, immediately after post-professional treatment, and at day 60 using the Schiff scale and a Visual Analog Scale (VAS).

**Results:**

Immediately after professional oxalate treatment, the overall mean Schiff and VAS score decreased 25.6% and 22.4% from baseline, respectively (*p* ≤ 0.001 for both). At day 60, further reductions in both mean scores were seen in both groups. There were no significant differences between the groups at day 60. All treatments were well tolerated.

**Conclusions:**

In subjects treated with oxalates for dentinal hypersensitivity, both stannous fluoride and sodium fluoride dentifrices are well tolerated, are feasible for routine use, and do not detract from the desensitizing effects of an in-office and at-home oxalate combination treatment regimen.

**Clinical relevance:**

Either stannous fluoride or sodium fluoride dentifrices can be recommended to dentinal hypersensitivity patients who undergo professional oxalate treatment.

## Introduction

Dentinal hypersensitivity, characterized by short, sharp pain from exposed dentin in response to thermal, evaporative, tactile, osmotic, or chemical stimuli [1], is a widespread clinical problem [2, 3]. Pain arises from altered fluid flow in dentinal tubules in response to temperature, desiccation, or osmotic balance fluctuations, resulting in nociceptor activation in the pulp/dentin border area [4]. Hypersensitive teeth have about 8 times the number of tubules per unit area than non-sensitive teeth, and tubule diameters are about twice as wide in hypersensitive teeth than in non-sensitive teeth [5, 6].

Common treatments for dentinal hypersensitivity rely on the principle of tubular occlusion [7, 8]. Specifically, professionally applied oxalates have been shown to deposit crystals at a depth of approximately 20 μm [9, 10], yielding a reduction in the dentinal fluid flow rate by 34.8% immediately after application [11, 12]. Professionally applied oxalate preparations have been shown to be effective desensitizing agents, in some cases with immediate onset of action [13–15]. Patient-applied oxalate preparations have also been investigated, with encouraging results. One randomized, controlled study examined the efficacy of a daily potassium oxalate mouthrinse versus a placebo mouthrinse for dentinal hypersensitivity and found significantly greater improvements at week 4 in the experimental group than in the placebo group [16]. A second placebo-controlled study compared the efficacy of professionally applied versus patient-applied dipotassium oxalate monohydrate dental strips at 30 min, week 4, and week 8 [17]. Both types of strip application produced significant improvements in dentinal hypersensitivity at all time points.

The desensitizing effects of oxalate treatment, both in-office and at-home, may last for several weeks to several months after application [13, 18–20]. Oxalate crystal deposits in dentinal tubules seem to be relatively resistant to dissolution by salivation, brushing, dentifrices, and acids [19–22]. However, the effect of oral hygiene upon the durability of the clinically relevant desensitization seen after oxalate treatment is not yet well understood. For example, dentifrices containing stannous fluoride have been shown to produce clinically meaningful desensitizing effects with regular use due to their ability to form a tin-rich layer on the surface [23–26]. A proportion of subjects with dentinal hypersensitivity may be treated with in-office or at-home oxalate preparations in combination with at-home use of a dentifrice containing stannous fluoride. There is little evidence as to what effect dentifrices might have (additive, no effect, or inhibitory) upon the durability of desensitization produced by the oxalate treatment. Therefore, the current study was designed to compare the effects of two different dentifrices, one containing stannous fluoride, and one containing sodium fluoride, on dentinal hypersensitivity following use of an oxalate-based in-office plus at-home regimen for tubular occlusion.

## Materials and methods

### Study objective

The objective of the study was to compare the effects of two different dentifrices on dentinal hypersensitivity following use of an oxalate-based in-office plus at-home regimen for tubular occlusion. Institutional review and approval were obtained from Schulman Associates IRB (201404679). After institutional review, subjects were recruited in August 2014 from a general dental practice (Fresno, CA, USA). All subjects provided written, informed consent. The study was conducted in compliance with the International Conference on Harmonization’s Good Clinical Practice Consolidated Guidelines and was registered with clinicaltrials.gov (NCT02221349).

### Subjects

Eligibility was limited to generally healthy adults 18 years of age or older. Subjects with at least one tooth with a positive air sensitivity response were eligible for this study; however, patients with dentinal hypersensitivity because of caries were excluded. Other exclusion criteria were allergy to aqua, glycerin, cellulose gum, dipotassium oxlate, carbomer, sodium hydroxide, sodium benzoate, and/or potassium sorbate; pregnancy or nursing; severe periodontal disease; active treatment for periodontitis; fixed facial orthodontic appliances; or a history of kidney stones. Enrolled subjects were required to refrain from dental prophylaxis or any elective dentistry during the study period.

### Assessments and outcomes

Dentinal hypersensitivity was assessed using the air blast challenge. Briefly, the challenge involves a one-second application of air from a standard dental unit syringe that is delivered onto a single target tooth; this is perceived as painful for subjects with dentinal hypersensitivity [27]. The two primary outcome measures in this study were the Visual Analog Scale (VAS) and the Schiff Index given in response to the air blast challenge on sensitive teeth. The examiner recorded a score on the Schiff scale [28] for each tooth tested (Table 1). Immediately thereafter, the examiner asked the subject to look at a VAS and to indicate the level of pain experienced because of the challenge. On the VAS, 0 is “no pain at all”, and 100 is “the worst tooth pain ever before experienced”.

**Table 1:** The Schiff Index Sensitivity Scale

Score	Criteria
0	Subject does not respond to stimulus
1	Subject responds to stimulus, but does not request discontinuation of stimulus
2	Subject responds to stimulus and requests discontinuation of stimulus or moves away from stimulus
3	Subject responds to stimulus, considers stimulus to be painful, and requests discontinuation of the stimulus

### Study design

This was a practice-based, randomized, controlled, double-blind, pilot study. Enrolled subjects participated in two study visits. At the first visit, subjects gave informed consent, and then were interviewed to determine their demographic information and personal medical history. Each subject then received a comprehensive clinical examination of the oral and perioral regions, including the hard and soft tissues. After the examination, the air blast challenge was conducted to identify up to two target teeth with dentinal sensitivity, defined as a Schiff score of ≥ 1, in different quadrants. Subjects were also asked to select a VAS score after each air blast challenge. Schiff scores and VAS scores were recorded as baseline values. Subjects were stratified based on baseline Schiff score, baseline VAS score, age, and gender. Within strata, subjects were randomly assigned to one of two groups using block randomization via an encoded program supplied by the study sponsor.

After randomization, all subjects were treated at the first study visit with a marketed 3% oxalate/potassium salt solution (Super Seal® Desensitizer, Phoenix Dental, Inc., Fenton, Michigan) on up to two identified target teeth. The oxalate/potassium salt solution was professionally applied by the examiner according to the manufacturer’s instructions for use. Immediately following the sensitivity treatment, the examiner performed the air blast challenge to the treated teeth, followed by subject self-assessment of pain using the VAS and examiner’s assessment of clinical sensitivity using the Schiff Index.

Next, subjects received an at-home dental hygiene kit box corresponding to their randomly assigned group. All investigational products were overlabeled for blinding and were provided by The Procter & Gamble Co., Cincinnati, OH, USA. The blinded kit boxes contained the randomly assigned dentifrice (the experimental variable in the study) plus sensitivity strips and a soft, manual toothbrush. The test product was either Crest® Pro-Health dentifrice (0.454% stannous fluoride) or Crest® Cavity Protection dentifrice (0.243% sodium fluoride). The products common to all subjects included a package of six sensitivity strips similar to those that have been marketed for at-home use (each 1 cm × 3 cm, gel containing 3.14% potassium oxalate) and an Oral-B® Indicator soft, manual toothbrush.

After receiving the appropriate kit box, subjects were instructed to use the test dentifrice and the soft, manual toothbrush in place of their normal oral hygiene routine and to bring all products back with them at the second study visit. Subjects were also instructed on how to apply the sensitivity strips. Strips were to be applied onto the facial surfaces of sensitive teeth for a duration of 10 min. Subjects were told that the test strips could be applied to up to two teeth, up to three times per tooth, throughout the study. Strip use was ad lib and unsupervised, which is consistent with typical at-home use of over-the-counter desensitizing products.

At the second study visit, which occurred at day 60, a comprehensive oral examination was conducted to evaluate the oral and perioral region, including hard and soft tissues. The air blast challenge was then performed to the teeth that had been treated at visit 1, followed by subject self-assessment of pain using the VAS and examiner’s assessment of clinical sensitivity using the Schiff Index. All subjects were asked how many sensitivity strips had been used (self-report).

### Adverse events

An adverse event was defined as any unfavorable or unintended sign, symptom, or disease that appeared or worsened in a subject during the study period. Adverse events were collected from examination and interview.

### Statistical analysis

A sample size of 15 subjects per group in this pilot study yielded 80% power to detect an effect size of 0.78 versus baseline, and between-group differences of 1.1, using two-sided testing at 5% significance levels.

Standard summary statistics of the demographic data and safety AE data were performed by group and overall. Sensitivity scores were averaged amongst target teeth for each subject at each assessment, before and after treatment. For both Schiff and VAS scores, changes versus baseline and change versus post-professional-treatment (PPT) baseline were tested using a 2-sided paired difference *t* test. To compare treatment groups, the mean difference was estimated using an analysis of covariance with PPT baseline as a covariate. Statistical comparisons were two-sided using a 5% significance level.

## Results

### Baseline demographics and study participation

The study population consisted of 30 subjects (93% female) with a mean age of 43.2 years (range, 19–66 years). The experimental and control groups were well balanced (*p* ≥ 0.36) for age and gender (Table 2).

**Table 2:** Baseline demographics

Demographic/ statistic or category	0.454% stannous fluoride	0.243% sodium fluoride	Overall	*P* value
Age (years)				
Mean (SD)	44.9 (12.52)	41.5 (10.97)	43.2 (11.69)	0.435
Min.-Max.	19–66	22–62	19–66	
Sex				
Female	14 (93%)	14 (93%)	28 (93%)	0.999
Male	1 (7%)	1 (7%)	2 (7%)	

Of the 30 subjects, 29 completed the study; 1 subject in the stannous fluoride group withdrew from the study before the day 60 evaluation. All subjects who completed the study reported to the investigator that they had used all six sensitivity strips.

### Immediate response to professional treatment

At baseline, the overall mean (SD) Schiff score was 2.73 (0.469), and overall mean (SD) VAS score was 65.30 (13.81). After professional, in-office treatment with the oxalate/potassium salt solution (Table 3), the overall mean (SD) Schiff score decreased 25.6% from baseline (*p <* 0.0001), and the overall mean (SD) VAS score decreased 22.4% from baseline (*p* < 0.001).

**Table 3:** Overall Schiff Air Index scores and Visual Analog Scale scores at baseline and immediately post professional treatment (PPT) with a 3% oxalate/potassium salt solution

Treatment	*N*	Mean (SD) score at baseline	Mean (SD) change in score from baseline at PPT	Mean percent change in score from baseline at PPT	*P* value
Overall Schiff Air Index score	30	2.733 (0.469)	− 0.7 (0.64)	25.6%	< 0.0001
Overall Visual Analog Scale score	30	65.30 (13.81)	− 14.6 (17.71)	22.4%	< 0.001

*PPT* post professional treatment

### Schiff scores and VAS scores at day 60

After 60 days of at-home dentifrice regimen use, subjects in both the stannous fluoride regimen group and the sodium fluoride regimen group showed further improvements in dentinal hypersensitivity relative to PPT baseline Schiff scores (*p* < 0.03) (Table 4). The adjusted mean (SE) Schiff scores were 1.24 (0.222) in the stannous fluoride regimen group and 1.11 (0.214) in the sodium fluoride regimen group. Dentifrice groups did not differ significantly (*p* = 0.68) at day 60.

**Table 4:** Schiff Air Index scores and Visual Analog Scale scores immediately post professional treatment (PPT) with a 3% oxalate/potassium salt solution and at day 60 by treatment group

Endpoint	Group	*N*	Mean (SD) score at PPT	Adjusted mean (SE) score at day 60	*P* value (day 60 vs. PPT)	*P* value (between group comparison at day 60)
Schiff Air Index Score	Sodium fluoride	15	2.17 (0.645)	1.11 (0.214)	0.001	0.681
Schiff Air Index Score	Stannous fluoride	14	1.89 (0.859)	1.24 (0.222)	0.029	
Visual Analog Scale Score	Sodium fluoride	15	59.13 (19.083)	38.85 (5.497)	0.03	0.875
Visual Analog Scale Score	Stannous fluoride	14	40.75 (21.889)	37.52 (5.709)	0.287	

*PPT* post professional treatment

For VAS scores, further improvements in dentinal hypersensitivity were demonstrated on day 60 in both the stannous fluoride regimen group (directional) and the sodium fluoride regimen group (*p* < 0.03) relative to PPT baseline scores (Table 4). The adjusted mean (SE) VAS scores were 37.52 (5.709) in the stannous fluoride regimen group and 38.85 (5.497) in the sodium fluoride regimen group. There was no statistically significant difference between the groups at day 60 (*p* = 0.875).

In this pilot study, 25 subjects (83%) exhibited appreciable air sensitivity (Schiff > 2) at baseline. Individual response to treatment varied (Table 5), but after 60 days, only one subject in the sodium fluoride group continued to exhibit this severe response. Self-reported pain using VAS showed similar improvements, and by group, the two endpoints were well-correlated (*r* > 0.70, *p* < 0.01). Only two subjects (one in each group) failed to exhibit reductions in either clinical or self-perception of sensitivity at study completion.

**Table 5:** Sensitivity by subject, endpoint, and visit

Sodium fluoride				Stannous fluoride			
Schiff Index		Visual Analog Scale		Schiff Index		Visual Analog Scale	
Baseline	Day 60	Baseline	Day 0	Baseline	Day 60	Baseline	Day 60
3	0	84	1.5	3	1.5	72	61
3	0	57.5	40.5	3	0.5	45.5	12.5
3	0.5	65.5	46	3	2	72.5	24.5
2	1	66	49.5	3	1	70	50
2	1	56	5	1.5	0	29.5	14
3	2	73	56.5	3	1.5	80	37
3	0.5	85.5	36	3	0	62.5	24.5
1.5	0.5	35	10	3	1	69	40.5
3	1.5	65	57.5	3	0.5	83.5	12.5
2.5	2	48.5	53	3	1	54	30
3	2	85	56.5	3	2	70	48
3	1	75	57.5	2.5	.	63.5	.
3	2	67.5	74	3	3	59.5	66.5
2.5	2.5	51.5	65	3	1.5	71.5	42.5
2	0.5	75.5	12	2.5	1.5	65.5	24

### Safety

The study regimens were well tolerated. One subject in the stannous fluoride group reported mild irritation and tooth sensitivity that resolved during the at-home usage period.

## Discussion

There is an unmet need for dentinal hypersensitivity regimens that are simultaneously effective, durable, and acceptable to subjects. For example, a recent, multi-center study from the National Dental Practice-Based Research Network examined outcomes after 8 weeks for 1862 subjects with dentinal hypersensitivity. Subjects were recruited by 171 dentists and were treated per the recruiting dentists’ desired protocols. These protocols could include dentist-provided treatments (glutaraldehyde/hydroxyethyl methacrylate compounds, oxalates, and bonding agents), dentists’ advice and counseling regarding oral habits and diet, and patient-applied fluoride toothpaste. Strikingly, after 8 weeks of treatment, 40% of subjects reported no improvement in pain [29]. The current practice-based, randomized, controlled, double-blind, pilot study in adult subjects with dentinal hypersensitivity demonstrated that (1) in-office, professional oxalate treatment on day 1 resulted in an immediate 22% to 25% reduction in hypersensitivity as assessed by VAS scores and Schiff Air Index scores; (2) VAS scores and Schiff scores showed further improvements by day 60 with regular use of at-home regimens consisting of subject-applied oxalate strips as well as either a dentifrice containing either sodium fluoride or a dentifrice containing stannous fluoride; and (3) VAS scores and Schiff scores did not significantly differ by day 60 between the sodium fluoride and stannous fluoride groups. Importantly, both at-home regimens in this exploratory study were well tolerated by subjects and were feasible for long-term use.

It has been well demonstrated previously that oxalate preparations are effective and relatively durable. The beneficial desensitizing effects of professionally applied oxalate treatments are immediate and can last for several weeks to months after application [13, 18–20]. Still, the effects of professional oxalate treatments may not last the entire time between visits for routine prophylaxis, supporting the idea of at-home oxalate use. Previous studies of self-applied, at-home potassium oxalate dental strips demonstrated good desensitizing effectiveness immediately after treatment with significant benefits versus the placebo group at week 4 post-treatment [17]. In the current pilot study, we combined two oxalate treatment options, both professionally applied and patient-applied. This approach has not yet been rigorously studied nor reported in the literature. Statistically and clinically significant reductions in mean Schiff scores and mean VAS scores were evident immediately after professional oxalate treatment, and scores continued to improve over time with the at-home use of oxalate dental strips plus a dentifrice containing either stannous fluoride or sodium fluoride.

At day 60, there were no significant differences in either Schiff scores or VAS scores between the stannous fluoride and sodium fluoride groups, suggesting that the two dentifrices did not overtly impact the sensitivity relief from the combined oxalate treatment regimen. It is interesting that stannous fluoride dentifrices have been shown by some SEM studies to create a tin-rich protective layer, occluding dentinal tubules [30–32]. It has therefore been hypothesized by other authors that stannous fluoride dentifrices may complement the occluding crystal deposits produced by an oxalate regimen. Numerous randomized controlled clinical trials have shown a significant desensitizing advantage benefit for stannous fluoride versus sodium fluoride dentifrice [25, 26, 33–37] due to stannous fluoride’s ability to create a tin-rich protective layer [30–32].

In our study, the effect of the combined oxalate treatments was considerable and may have minimized any incremental dentifrice contribution. Indeed, in vitro research has demonstrated that potassium oxalate strips were significantly more effective at reducing fluid movement through human dentin than numerous occluding ingredients used in rinses and dentifrices, including stannous fluoride [38]. It is worth noting that the effectiveness of active ingredients can be affected by the product’s delivery system, contact time, and formulation, so the effectiveness results for one product cannot necessarily be generalized to another product, even if both products contain the same active ingredients. Another limitation of our study was randomization with stratification based on baseline Schiff and VAS scores. Because we did not anticipate that the average response to professional treatment would differ between the groups, the average response to professional treatment was not used when stratifying subjects before randomization. When considering future research on the effects of various dentifrices upon dentinal hypersensitivity after professional oxalate treatment, this pilot study (with its limitations) will be helpful to inform the development of future study protocols. Still, our exploratory study demonstrates an important finding that a stannous fluoride dentifrice is just as safe and tolerable for long-term use as is a sodium fluoride dentifrice in “at-risk” subjects with dentinal hypersensitivity who have been treated with oxalate preparations. This is a clinically relevant finding, as stannous fluoride dentifrices have been shown in many clinical studies to provide superior anti-gingivitis benefits [39–41] and superior protection against acid erosion [42, 43] as compared with control dentifrices containing sodium fluoride.

## Conclusion

This pilot, practice-based research demonstrates that, in subjects treated with oxalates for dentinal hypersensitivity, both stannous fluoride and sodium fluoride dentifrices are well tolerated, are feasible for routine use, and do not detract from the desensitizing effects of an in-office and at-home oxalate combination treatment regimen.

## Abbreviations

VAS: Visual Analog Scale
